# Tool Cutting Force Prediction Model Based on ALO-ELM Algorithm

**DOI:** 10.1155/2022/1486205

**Published:** 2022-09-26

**Authors:** Hongna Zhang

**Affiliations:** College of Engineering, Inner Mongolia University for Nationalities, Tongliao 028000, Inner Mongolia, China

## Abstract

Aiming at the problems of low learning efficiency, slow convergence speed, and low prediction accuracy of traditional data-driven model applied to tool cutting force prediction, a prediction method of tool cutting force based on ant lion optimizer (ALO) extreme learning machine (ELM) is proposed. ALO was used to improve the weights of input layer and hidden layer of ELM, so as to improve its prediction accuracy. The tool cutting force prediction models were established by using ALO-ELM, ELM, BP (backpropagation) neural network, and support vector machine, respectively. The experimental results show that the mean square error, mean absolute percentage error, and mean absolute error of ALO-ELM prediction model are 0.9911%, 0.0011%, and 1.0863%, respectively, which are far lower than the other three prediction models. ALO-ELM prediction model has stronger prediction accuracy and generalization ability, which can be effectively applied to the prediction of cutting force.

## 1. Introduction

In modern manufacturing, as an important indicator that directly affects workpiece processing—cutting force, it is closely related to product quality and production cost [1]. However, since there are many factors affecting the cutting force, and there is a highly complex relationship between it and the cutting force, it is difficult to predict the cutting force [2]. Scholars at home and abroad often use the empirical formula method and the physical analysis method to model the cutting force, but the two methods also have the following limitations. First, most of the parameters involved in the model need to be determined through experiments; second, the driving of the mathematical model must be based on domain expert knowledge, so it takes a lot of time, manpower, and material resources.

In this case, a data-driven model can be the solution. The scheme is based on the monitoring data provided by the sensor and realizes the prediction of the cutting force of the tool by fitting the test data, emphasizing the modeling according to the historical data. For the problem of predicting tool cutting force, scholars have also tried to solve the problem of tool cutting force prediction through data-driven models. For example, Hashemitaheri et al. established comparative models based on support vector machine and Gaussian process regression, respectively, to predict cutting force [3]; Wang and Chao proposed a prediction model of cutting force based on combination algorithm [4]; Xiang and Zhang established a prediction model of cutting force through WOA-Kriging algorithm [5].

Although typical data-driven models (such as backpropagation (BP) neural network and support vector machine) have good nonlinear approximation ability and strong generalization ability, this model also has problems such as low learning efficiency, slow convergence speed, and easy to fall into local optimum. Extreme learning machine (ELM), proposed by Huang et al. [6] in 2004, is a single-hidden-layer feedforward neural network (single-hidden-layer feedforward neural networks) machine learning algorithm. It makes up for the shortcomings of slow learning speed and gradient descent of neural networks, has the characteristics of fast training speed and good generalization performance [7], and has been successfully used in classification [8], regression prediction [9], and other fields. However, since the ELM randomly generates the weights of the input layer and the hidden layer, the trained ELM model cannot achieve the optimal performance, which will affect the generalization performance and stability of the ELM. To ensure ELM better model, accuracy can only be achieved by increasing the number of neurons in the hidden layer, and increasing the number of neurons in the hidden layer will increase the running time of the model and reduce the efficiency. After 2015, Genetic Algorithm (GA) [10], Particle Swarm Optimization (PSO) [11], Grey Wolf Optimizer (GWO) [12], Simulated Annealing The emergence of swarm intelligence optimization algorithms (Swarm Intelligence, SI) such as Annealing, SA) [13] has made this problem a better solution. Because the swarm intelligence algorithm has the characteristics of simple operation, fast convergence speed, and good global convergence, it has become an ideal method for scholars to optimize ELM parameters to improve model performance. Also, ant lion optimization (ALO) is also a swarm intelligence optimization algorithm, although it was only proposed in 2015 [14]. However, it is widely used in the engineering field because of its characteristics of less parameters to be set, good convergence, and high robustness. Compared the prediction performance of the PSO algorithm and the ALO algorithm on the same problem. The experimental results show that ALO algorithm has better performance in optimization accuracy, global search ability, and parameter setting than PSO algorithm. Therefore, ALO algorithm was used in this paper to automatically optimize the parameters of ELM.

Based on the above content, this paper proposes a tool cutting force prediction method based on ALO-ELM. First, the principles and operation steps of ELM and ALO algorithms are introduced, and then, ALO-ELM, ELM, BP neural network, and SVM are used to establish a tool cutting force prediction model. Root mean square error (RMSE), mean absolute percentage error (MAPE), and mean absolute error (MAE) were used to evaluate the prediction effects of the four models. The experimental results prove that the classification model based on ALO-ELM has higher classification accuracy.

## 2. Theoretical Overview of the ALO-ELM

### 2.1. Extreme Learning Machine

As a relatively new data-driven method (compared to artificial neural network—ANN—and support vector machine—SVM), extreme learning machine adopts an efficient single-hidden-layer feedforward neural network [6]. This is different from the traditional backpropagation algorithm ANN, which provides a way to solve the output weights through the least-squares method instead of iteration. Since the input layer weights and hidden layer thresholds are random, and the output has a unique least-squares solution, the ELM model is able to solve regression (or classification) problems in a short time. At the same time, relying on the Moore-Penrose generalized inverse [6], ELM can solve the problem that the traditional backpropagation algorithm tends to be locally optimal.

**Figure 1:**
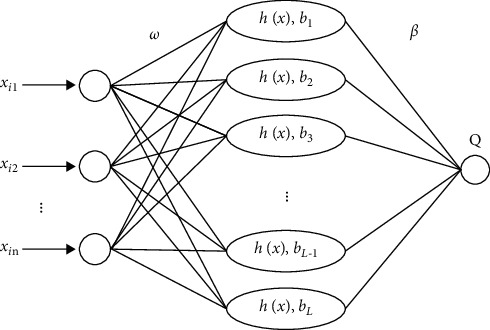
ELM network structure.

Generally, the ELM network structure consists of an input layer, an output layer, and a hidden layer. Input layer weights and hidden layer thresholds are used to establish connections between two adjacent layers. In addition, in the ELM model, the weights of the input layer and the thresholds of the hidden layer are randomly generated. Therefore, the parameters that need to be manually set are only the activation function and the number of neurons in the hidden layer. Suppose there are *Q* different training samples (*X*_*i*_, *Y*_*i*_) ∈ *R*^*n*^ × *R*^*m*^, if *L* is the number of neurons in the hidden layer, the standard feedforward neural network can be described as follows:

(1) $$\begin{array}{c} \sum_{i=1}^{L}\beta_{i}h\left(w_{i}\cdot x_{i}+b_{i}\right)=Q_{j},j=1,\ldots ,N, \end{array}$$

where *w*_*i*_=[*w*_*i*1_, *w*_*i*2_,…,*w*_*in*_]^*T*^ is the weight connecting the input layer node and the ith hidden layer neuron; *β*_*i*_=[*β*_*i*1_, *β*_*i*2_,…,*β*_*in*_]^*T*^is the weight connecting the ith hidden layer neuron and the output layer node; *b*_*i*_ is the threshold of the ith hidden layer neuron; *Q*_*j*_=[*Q*_*j*1_, *Q*_*j*2_,…,*Q*_*jn*_]^*T*^ is the output of the network; *h*(*x*) represents the activation function, and the sigmoid activation function is used in this paper. The network structure of ELM is shown in Figure 1.

If the output matrix of ELM is set to *H*, the training result of the model and the expected output result *y*_j_ can be close to zero error after a certain training time, and the expression is

(2) $$\begin{array}{c} \sum_{i=1}^{L}\beta_{i}h\left(w_{i}\cdot x_{i}+b_{i}\right)=y_{j},j=1,\ldots ,N. \end{array}$$

Equation (3) can also be converted into the following matrix form:

(3) $$\begin{array}{c} H\beta =Y. \end{array}$$

In the formula, *H* represents the output matrix obtained from the hidden layer, and its expression is

(4) $$\begin{array}{c} H=\left(\begin{array}{c} h\left(x_{1}\right)\\ \vdots \\ h\left(x_{N}\right) \end{array}\right)={\left(\begin{array}{ccc} h\left(w_{1}\cdot x_{1}+b_{1}\right) & \cdots & h\left(w_{L}\cdot x_{1}+b_{L}\right)\\ \vdots & \cdots & \vdots \\ h\left(w_{1}\cdot x_{N}+b_{1}\right) & \cdots & h\left(w_{L}\cdot x_{N}+b_{L}\right) \end{array}\right)}_{N\times L}. \end{array}$$

The expression of the expected output weight matrix *Y* is

(5) $$\begin{array}{c} Y=\left(\begin{array}{c} y_{1}^{T}\\ \vdots \\ y_{N}^{T} \end{array}\right)=\left(\begin{array}{ccc} y_{11} & \cdots & y_{1m}\\ \vdots & \; & \vdots \\ y_{N1} & \cdots & y_{Nm} \end{array}\right). \end{array}$$

The purpose of ELM training is to calculate the minimum value of the error of *Hβ* − *Y*′. When the activation function is infinitely differentiable, the smallest $\hat{\beta }$ can be determined according to the least-squares method:

(6) $$\begin{array}{c} \underset{\beta }{\min}\left\|H\beta -Y^{\prime }\right\|. \end{array}$$

Final results are as follows:

(7) $$\begin{array}{c} \hat{\beta }=H^{+}Y, \end{array}$$

where *H*^+^ is generalized inverse of H, and $\hat{\beta }$ is the weight matrix of the output layer.

### 2.2. Antlion Algorithm

Antlion algorithm is a novel natural heuristic algorithm proposed by Mirjalili et al. in 2015 [14]. It is based on modeling the hunting mechanism of antlions in nature and includes five main steps, namely random walk of ants, construction of traps, entrapment of ants in traps, capture of ants, and reconstruction of traps. It has the advantages of less adjustment parameters and better optimization accuracy. This section will introduce the mathematical model of the antlion algorithm.

#### 2.2.1. Ant Random Walk

Ants move through the search space affected by antlion traps by random walks and change their positions according to the following equation:

(8) $$\begin{array}{c} X\left(t\right)=\left[0,cs\left(2r\left(t_{1}\right)-1\right),cs\left(2r\left(t_{2}\right)-1\right),\ldots ,cs\left(2r\left(t_{n}\right)\right)-1\right]. \end{array}$$

Among them, cs represents the calculation of the cumulative sum, *T* represents the maximum number of iterations, *t* is the current iteration number, and *r*(*t*) represents the random function, which is defined as follows:

(9) $$\begin{array}{c} r\left(t\right)=\left\{\begin{array}{c} \begin{array}{ccc} 1 & \text{if} & \text{rand}>0.5 \end{array},\\ \begin{array}{ccc} 0 & \text{if} & \text{rand}<0.5, \end{array} \end{array}\right) \end{array}$$

where rand is a uniformly distributed random number generated in the interval [0, 1]. At the same time, in order to ensure that the random walks of all ants fall within the boundary of the search space, the normalization process is carried out using the following formula:

(10) $$\begin{array}{c} X_{i}^{t}=\frac{\left(X_{i}^{t}-a_{i}\right)\times \left(d_{i}^{t}-c_{i}^{t}\right)}{\left(b_{i}-a_{i}\right)}+c_{i}^{t}, \end{array}$$

where *a*_*i*_ and *b*_*i*_ are the minimum and maximum values of the random walk for the *i*th variable, and *c*_*i*_^*t*^ and *d*_*i*_^*t*^ are the minimum and maximum values for the *t*-th iteration of the *i*th variable.

#### 2.2.2. Build a Trap

The roulette wheel is used to simulate the hunting ability of the antlion, and the ALO algorithm selects the most suitable antlion through the roulette wheel to make the probability of catching ants higher.

#### 2.2.3. Trapped in an Antlion Trap

The random walk of the ants will be affected by the antlion trap location, which is mathematically explained using the following formula:

(11) $$\begin{array}{c} c_{i}^{t}={\text{Antlion}}_{j}^{t}+c^{t}\\ d_{i}^{t}={\text{Antlion}}_{j}^{t}+d^{t}. \end{array}$$

Among them, *c*_*t*_ and *d*_*t*_ are the minimum and maximum values of all variables, and Antlion_*j*_^*t*^ represents the position of the *j* antlion obtained in the tth iteration. The variables *c* and *d* together define the roaming behavior of the ants within a trap constructed by the selected antlion.

#### 2.2.4. Ant Sliding to Antlion

When an ant walks into the antlion's trap, in order to slide the ant towards the antlion, the antlion shoots sand outward until the trapped ant slips down. The mathematical model of the above operation can be realized by adaptively reducing the hypersphere radius of the ant random walk, and the formula is

(12) $$\begin{array}{c} c^{t}=\frac{c^{t}}{I}\\ d^{t}=\frac{{\text{d}}^{t}}{I}. \end{array}$$

In the formula, *I*=10^*w*^*t*/*T*., *t* is the current number of iterations, *T* is the maximum number of iterations, *w* is a constant defined based on the current number of iterations, which can adjust the accuracy of the search, expressed as

(13) $$\begin{array}{c} w=\left\{\begin{array}{c} 2,t>0.1T\\ 3,t>0.5T\\ 4,t>0.75T\\ 5,t>0.9T\\ 6,t>0.95T. \end{array}\right) \end{array}$$

#### 2.2.5. Catch the Ants and Rebuild the Trap

The final stage of antlion hunting is to capture ants that slip to the bottom of the pit, and then, the antlion must update its position to the latest position of the hunted ants through equation (2.14) to increase its chances of catching other ants.

(14) $$\begin{array}{c} {\text{Antlion}}_{j}^{t}={\text{Ant}}_{i}^{t}\text{ if }f\left(Ant_{i}^{t}\right)>f\left({\text{Antlion}}_{j}^{t}\right). \end{array}$$

Among them, Ant_*i*_^*t*^ is the position of the *i*th ant in the *t*-th iteration, and *f*(·) is the fitness function.

#### 2.2.6. Elitism

The elite antlion is the optimal solution obtained in each iteration, which affects the motion of all ants during the iteration. Therefore, each ant will randomly walk around the antlion and elite antlion chosen by the roulette wheel, and the process can be modeled as

(15) $$\begin{array}{c} {\text{Ant}}_{i}^{t}=\frac{R_{A}^{t}+R_{E}^{t}}{2}, \end{array}$$

where *R*_*A*_^*t*^is the random walk of the antlion selected by the roulette principle in the *t*-th iteration, and *R*_*E*_^*t*^ is the random walk of the ants around the elite antlion in the *t*-th iteration.

## 3. Construction of Cutting Force Prediction Model Based on ALO-ELM

Since the input layer weights and hidden layer thresholds of the extreme learning machine are randomly generated, in order to improve the extreme learning machine and improve the accuracy of the model, ALO is used for the input layer weights *w*_*i*_=[*w*_*i*1_, *w*_*i*2_,…,*w*_in_]^*T*^ is optimized with the hidden layer threshold *b*_*i*_. In order to quantitatively analyze the accuracy of the tool cutting force prediction model, the statistical index mean square error (MSE) is used as the individual fitness to evaluate the prediction results, and its expression is as follows:

(16) $$\begin{array}{c} \text{MSE}=\frac{1}{n}\sum_{i=1}^{n}{\left(y_{i}-{\hat{y}}_{i}\right)}^{2}. \end{array}$$

Among them, *n* is the number of sample data, *y*_*i*_ is the actual tool cutting force, ${\hat{y}}_{i}$ is the predicted tool cutting force, and the prediction result with smaller MSE will be considered better.

The relevant optimization function can be expressed as

(17) $$\begin{array}{c} \min F\left(w,b\right)\\ \text{s}.\text{t}.\left\{\begin{array}{c} w\in \left(-1,1\right)\\ b\in \left(-1,1\right). \end{array}\right) \end{array}$$

**Figure 2:**
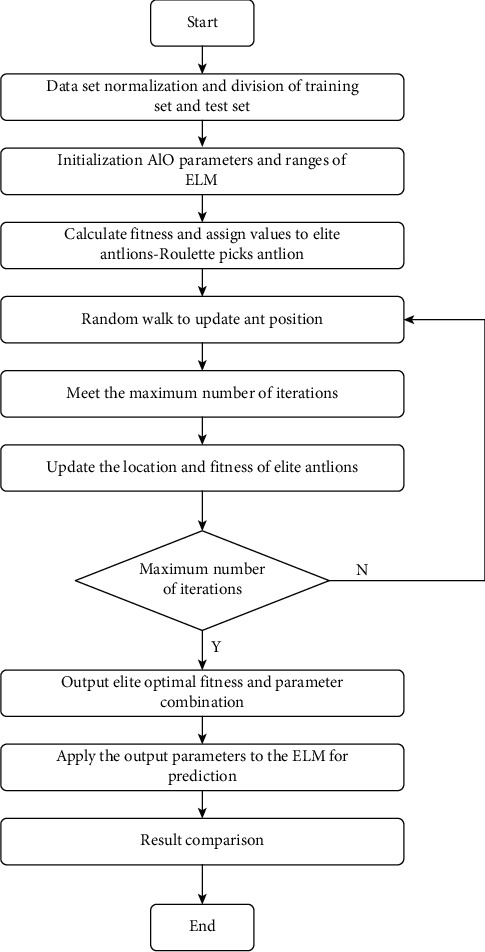
Predictive model workflow.

The specific workflow of the extreme learning machine tool cutting force prediction model based on ALO-ELM is as follows (Figure 2):

1. Data preprocessing: analyze and select the main factors that affect the cutting force of the tool according to relevant theories, determine the input and output parameters of the model, and normalize the data set to eliminate dimensional differences.
2. Prediction model establishment and data analysis: divide the training set and test set according to the ratio of 8 : 2, and then evaluate the fitness value of the antlion based on the MSE value and determine the antlion with the minimum MSE value through multiple iterations. The information corresponding to the input layer weight and the hidden layer threshold carried by the antlion is the optimal ELM input weight parameter and the hidden layer threshold parameter. After this, the model will be used to predict the test data.
3. Validation of the prediction model: input test set data, and compare the prediction results with the measured values to evaluate the accuracy of the prediction model.

## 4. Experimental Simulation and Result Analysis

### 4.1. Experimental Program

Considering that the cutting force of tooth cutting is affected by the workpiece speed, tool speed, feed, and depth of cut in practice, the four cutting parameters are selected as variables for testing.

The machine tool used in the tooth cutting experiment is a vertical CNC tooth cutting machine; the workpiece material is the finished product with 83 teeth, and the material is 45# steel; the tool has 40 teeth, and the material is GU20; the measuring equipment for the cutting force is a Kistler 9171A rotary dynamometer.

The attribute information of the 25 groups of data measured in this experiment is shown in Table 1.

According to step 1 in the tool cutting force prediction model, the sample data set is normalized, and finally, 80% of the total number of samples is selected as the training data set by uniform random selection, and the remaining 20% is used as the test data set.

### 4.2. Parameter Settings

**Figure 3:**
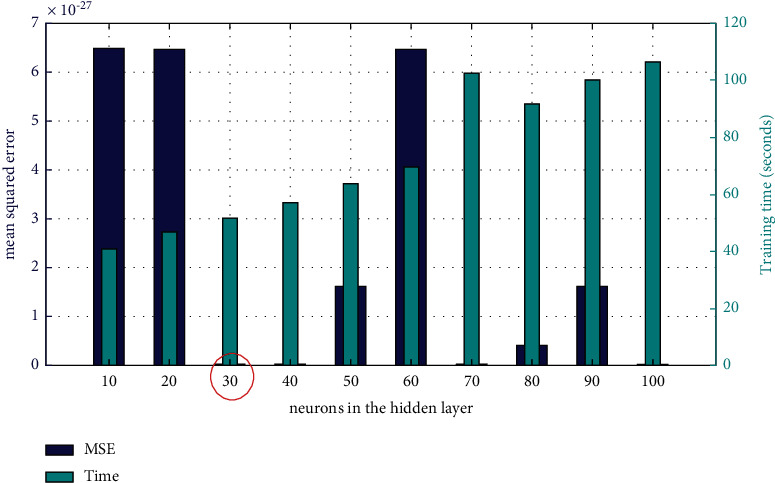
MSE and training time of ALO-ELM under different numbers of neurons in the hidden layer.

The ALO-ELM prediction model mainly involves three parameters: the number of neurons in the hidden layer, *L*, the population size of antlions and ants, *N*, and the maximum number of iterations, *T*. First, in order to determine the optimal number of hidden layer neurons, the interval of the number of hidden layer neurons was set as [10, 100], the population size of antlions and ants was set as 100, and the maximum iteration was set as 100. Also, the results shown in Figure 3 are obtained by using MSE and training time analysis. When the number of neurons in the hidden layer is 30, its MSE is relatively small, and its training time is the smallest among all network structures with the same prediction accuracy, so the optimal number of neurons in the hidden layer is determined to be 30.

**Figure 4:**
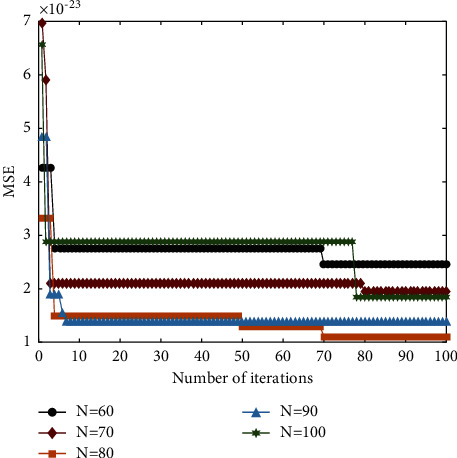
Iteration of ALO-ELM under different population sizes.

Second, the population size *N* was set to 60, 70, 80, 90, and 100, respectively, and the convergence of the prediction model under 100 iterations and different population sizes was calculated. The results are shown in Figure 4.

It can be seen from Figure 4 that under different population sizes, the MSE values of the ALO-ELM prediction model show a significant decrease with the increase of the number of iterations. At the same time, considering that the training time of the model will greatly increase with the expansion of the population size, it is necessary to comprehensively consider the training time and prediction accuracy. It can be seen from Figure 4 that when the population size *N* is set to 80, a good balance can be achieved between training time and prediction accuracy. At the same time, when the iteration reaches about 90 times, the fitness MSE of the prediction model remains basically stable. Therefore, in order to take into account the time factor, the maximum number of iterations *T* is set to 90.

### 4.3. Analysis and Comparison of Experimental Results

**Figure 5:**
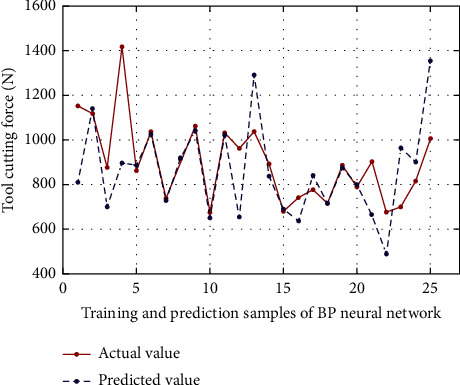
Comparison of predicted value and actual value of BP neural network.

**Figure 6:**
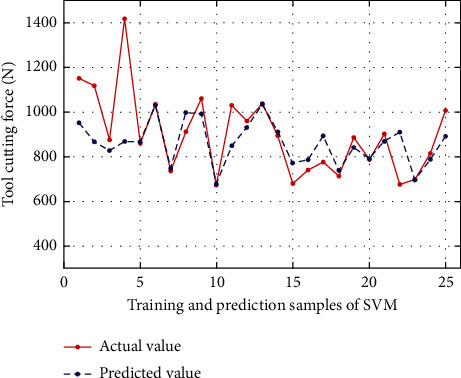
Comparison of SVM predicted value and actual value.

**Figure 7:**
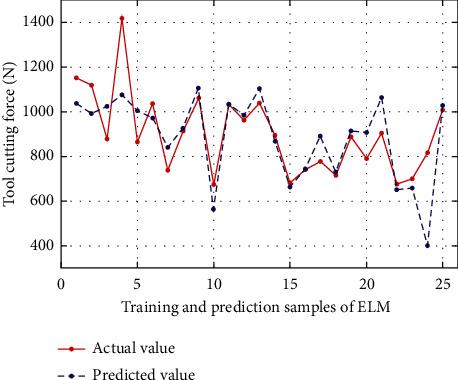
Comparison of ELM predicted value and actual value.

**Figure 8:**
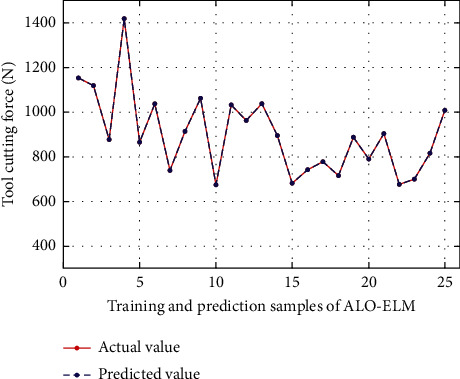
Comparison of ALO-ELM predicted value and actual value.

In order to verify the feasibility and superiority of ALO to optimize ELM in this paper, on the basis of ALO-ELM to predict cutting force, the prediction results of the ALO-ELM method in this paper are compared with the traditional ELM, BP neural network, and SVM for cutting force. And analyze the performance of each algorithm. At the same time, considering the randomness of the algorithm, under the same conditions, the simulation experiments of the above four algorithms are repeated many times, and the average value is taken. Second, the three performance indicators of mean absolute error (MAE), root mean square error (RMSE), and mean absolute percentage error (MAPE) are used to comprehensively evaluate the prediction effects of the three models. The calculation formulas are as follows:

(18) $$\begin{array}{c} MAE=\frac{1}{n}\sum_{i=1}^{n}\left|y_{i}-{\bar{y}}_{i}\right|,\\ MAPE=\frac{100}{n}\sum_{i=1}^{n}\left|\frac{y_{i}-{\bar{y}}_{i}}{y_{i}}\right|,\\ RMSE=\sqrt{\frac{1}{n}\sum_{i=1}^{n}{\left(y_{i}-{\bar{y}}_{i}\right)}^{2}}, \end{array}$$

where *n* is the total number of data samples, and *y*_*i*_ and ${\bar{y}}_{i}$ are the actual and predicted values, respectively. The prediction results of each model are shown in Figures 4–8, where samples 1 : 20 are training sets, and samples 21 : 25 are test sets. The prediction performance comparison of each model is shown in Table 2.

As can be seen from Table 2 and Figures 4–8, compared with BP neural network and SVM, the accuracy of ELM in predicting cutting force has been improved to a certain extent. Compared with the BP neural network, the mean square error RMSE, the RMSE, MAPE, and MAE values of ELM prediction model are numerically reduced by 84.4, 0.041, and 41.258, respectively, compared with BP neural network, and 45.01, 0.0003, and 5.643, respectively, compared with SVM. Therefore, to a certain extent, it shows the effectiveness of ELM in predicting the cutting force of the tool. At the same time, it is worth noting that the prediction effect of the ALO-ELM prediction model is the best, and the prediction results of the test set and training set have a higher degree of fitting than the actual value. At the same time, the mean square error RMSE, the mean absolute percentage error MAPE, and the mean absolute error MAE all show a significant reduction compared with ELM, which is enough to foresee the advantages of the swarm intelligence algorithm. It shows that it is feasible to use the antlion optimization algorithm to optimize the extreme learning machine in the prediction of tool cutting force.

## 5. Conclusion

In this paper, aiming at the problems of low learning efficiency, slow convergence speed, and low prediction accuracy of traditional data-driven model applied to tool cutting force prediction, a tool life prediction method based on ALO-ELM was proposed based on ELM. At the same time, this paper compares the model with the traditional ELM prediction model, BP neural network prediction model, and SVM prediction model. The results show that the prediction effect of this model is the most consistent with the actual cutting force compared with the model without algorithm optimization, and the accuracy is the highest. Therefore, it can provide a new method for the prediction of cutting force. In addition, the model is a black box model, so it has strong adaptability, and the parameters can be changed according to the actual conditions to achieve the prediction of different tools.

## Figures and Tables

**Table 1:** Experimental dataset.

Sample number	Workpiece speed (r/min)	Depth of cut *p* (mm)	Axial feed (mm/r)	Tool speed (r/min)	Cutting force (N)
1	247.5	0.1	0.1	450	674.5369
2	275	0.1	0.12	500	698.6415
3	330	0.1	0.15	600	715.0175
4	357.5	0.1	0.18	650	680.014
5	385	0.1	0.2	700	816.1648
6	247.5	0.15	0.12	450	737.7845
7	275	0.15	0.15	500	789.5252
8	330	0.15	0.18	600	885.8945
9	330	0.15	0.2	600	1030.643
10	385	0.15	0.1	700	741.0297
11	247.5	0.2	0.15	450	876.6236
12	275	0.2	0.18	500	1118.269
13	330	0.2	0.2	600	892.9726
14	357.5	0.2	0.1	650	903.3825
15	385	0.2	0.12	700	1006.926
16	247.5	0.25	0.18	450	1416.827
17	275	0.25	0.2	500	777.2387
18	330	0.25	0.1	600	961.5092
19	330	0.25	0.12	600	1151.608
20	385	0.25	0.15	700	1061.485
21	247.5	0.3	0.2	450	863.1338
22	275	0.3	0.1	500	675.6478
23	330	0.3	0.12	600	913.7286
24	357.5	0.3	0.15	650	1037.882
25	385	0.3	0.18	700	1036.097

**Table 2:** Performance comparison of prediction models.

Models	RMSE	MAPE (%)	MAE
BP	187.6374	0.1300	123.5699
SVM	148.2492	0.0893	87.9549
ELM	103.2370	0.0890	82.3118
ALO-ELM	0.9911	0.0011	1.0863

## Data Availability

The raw/processed data required to reproduce these findings cannot be shared at this time as the data also forms part of an ongoing study.

## References

[1] Grum J. (2008). Book review: fundamentals of machining and machine tools by G. Boothroyd and W.A. Knight. *International Journal of Microstructure and Materials Properties*.

[2] Wang G., Peng D., Qin X., Cui Y. (2012). An improved dynamic milling force coefficients identification method considering edge force. *Journal of Mechanical Science and Technology*.

[3] Hashemitaheri M., Mekarthy S. M. R., Cherukuri H. (2020). Prediction of specific cutting forces and maximum tool temperatures in orthogonal machining by Support Vector and Gaussian Process Regression Methods. *Procedia Manufacturing*.

[4] Wang Q. J., Chao L. I. (2013). A cutting force prediction based on combination algorithm. *Modular machine tool & automatic manufacturing technique*.

[5] Xiang Y., Zhang Q. (2020). Cutting force prediction mathematical model of titanium alloy based on WOA-Kriging algorithm. *Mechanical & Electrical Engineering Magazine*.

[6] Huang G. B., Zhu Q. Y., Siew C. K. Extreme learning machine: a new learning scheme of feedforward neural networks.

[7] Shiguo S. U. N., Zhenhua S. U. (2016). Research on the synthetical prediction method of landslip deformation. *Journal of Engineering Geology*.

[8] Huang G. B., Ding X., Zhou H. (2010). Optimization method based extreme learning machine for classification. *Neurocomputing*.

[9] Wang M., Chen H., Li H. (2017). Grey wolf optimization evolving kernel extreme learning machine: application to bankruptcy prediction. *Engineering Applications of Artificial Intelligence*.

[10] Fangcheng L., Liu Y. (2018). Short-term load forecasting based on optimized learning machine using improved genetic algorithm. *Journal of North China Electric Power University*.

[11] Shan P., Xinyi Y. (2017). Evolutionary extreme learning machine optimized by quantum-behaved Particle swarm optimization. *Journal of System Simulation*.

[12] Qiao W., Meng W. (2021). Estimation of lithium-ion battery SOC based on GWO-optimized extreme learning machine. *Energy Storage Science and Technology*.

[13] Sha L., Shi L. (2017). SA-ELM based method for reconstructing temperature distribution in acoustic tomography measurement. *CIESC Jorunal*.

[14] Mirjalili S. (2015). The ant lion optimizer. *Advances in Engineering Software*.

